# Pregnancy Incidence and Risk Factors among Women Participating in Vaginal Microbicide Trials for HIV Prevention: Systematic Review and Meta-Analysis

**DOI:** 10.1371/journal.pone.0077014

**Published:** 2013-10-10

**Authors:** Alfred Musekiwa, Evans Muchiri, Samuel O. M. Manda, Henry G. Mwambi

**Affiliations:** 1 School of Mathematics, Statistics and Computer Science, University of KwaZulu-Natal, Pietermaritzburg, South Africa; 2 Wits Reproductive Health & HIV Institute (WRHI), Faculty of Health Sciences, University of the Witwatersrand, Johannesburg, South Africa; 3 Biostatistics Unit, South African Medical Research Council, Pretoria, South Africa; University of Iowa Carver College of Medicine, United States of America

## Abstract

**Introduction:**

Pregnancy is contraindicated in vaginal microbicide trials for the prevention of HIV infection in women due to the unknown maternal and fetal safety of the microbicides. Women who become pregnant are taken off the microbicide during pregnancy period but this result in reduction of the power of the trials. Strategies to reduce the pregnancy rates require an understanding of the incidence and associated risk factors of pregnancy in microbicide trials. This systematic review estimates the overall incidence rate of pregnancy in microbicide trials and describes the associated risk factors.

**Methods:**

A comprehensive literature search was carried out to identify eligible studies from electronic databases and other sources. Two review authors independently selected studies and extracted relevant data from included studies. Meta-analysis of incidence rates of pregnancy was carried out and risk factors of pregnancy were reported narratively.

**Results:**

Fifteen studies reporting data from 10 microbicide trials (N=27,384 participants) were included. A total of 4,107 participants (15.0%) fell pregnant and a meta-analysis of incidence rates of pregnancy from 8 microbicide trials (N=25,551) yielded an overall incidence rate of 23.37 (95%CI: 17.78 to 28.96) pregnancies per 100 woman-years. However, significant heterogeneity was detected. Hormonal injectable, intra-uterine device (IUD) or implants or sterilization, older age, more years of education and condom use were associated with lower pregnancy. On the other hand, living with a man, history of pregnancy, self and partner desire for future baby, oral contraceptive use, increased number of unprotected sexual acts and inconsistent use of condoms were associated with higher pregnancy.

**Conclusions:**

The incidence rate of pregnancy in microbicide trials is high and strategies for its reduction are urgently required in order to improve the sample size and power of these trials.

## Introduction

The quest to identify effective HIV prevention interventions continues after more than three decades since the HIV/AIDS epidemic began. Anti-retroviral drugs (ARVs) have been shown to reduce heterosexual transmission of HIV [1]. Male condoms can reduce the risk of HIV infection by 80-90% when used correctly and consistently [2,3] and medical male circumcision has also been shown to reduce acquisition of HIV by heterosexual men by between 38% and 64% over 24 months [4]. However, HIV/AIDS remain a major public health problem with a global estimate of 2.5 million new HIV infections in 2011 alone [5]. Prevention of new HIV infections therefore remains the best method of controlling the epidemic. The burden of HIV is higher among women than men and since male condoms and medical male circumcision are male specific and usually beyond the control of women, HIV prevention interventions that are women-controlled have become a priority [6].

The majority of HIV prevention interventions targeting women are vaginal microbicides [6]. Beginning in 1987, there has been 14 vaginal microbicide trials for the prevention of heterosexual acquisition of HIV conducted mainly in Sub-Saharan Africa (Table 1) [7-20]. One of these trials [19] has just been completed and one is still ongoing [20]. Vaginal microbicides are compounds that can be applied inside the vagina to protect against acquisition or transmission of HIV during sexual intercourse [6]. These microbicide trials enroll non-pregnant HIV-negative sexually active women of reproductive age [21]. Although this target population group is at high risk of HIV infection, it is also at risk of pregnancy. It is known that these microbicide trials experience high rates of pregnancy despite the fact that participants receive family planning counseling and effective contraception during the trials [22]. Study protocols require that women who become pregnant be taken off the investigational product due to the unknown safety of the microbicides to both the mother and the fetus [21]. These drop-outs result in higher attrition rates which compromise the sample size, power, and interpretation of the microbicide trials [22]. Pregnancy is also undesired in the microbicide trials because women are at higher risk of HIV infection during pregnancy [23]. And also this may result in mother to child transmission of HIV. In order to improve eligibility criteria and develop strategies of reducing the undesired pregnancies for future trials, it is crucial to determine the risk factors for pregnancy in identical trial populations [22].

**Table 1:** Timeline of Microbicide Trials.

**Year**	**Microbicide**	**Trial Name**	**Country**	**Population**
1987-1990	N-9 Sponge	Kenya N-9 Sponge [7]	Kenya	Female Sex Workers
1994-1996	N-9 Film	FHI N-9 Film [8]	Cameroon	Female Sex Workers
1996-1998	N-9 Gel	Low-dose N-9 Gel Trial [9]	Kenya	Female Sex Workers
1996-2000	N-9 Gel	UNAIDS COL-1492 Trial [10]	South Africa, Thailand, Benin, Cotê d’Ivoire	Female Sex Workers
2004-2006	SAVVY (C31G) Gel	FHI SAVVY Trial/ Ghana [11]	Ghana	High Risk Women
2004-2006	SAVVY (C31G) Gel	FHI SAVVY Trial/ Nigeria [12]	Nigeria	High Risk Women
2004-2007	Cellulose Sulphate Gel	FHI CS Trial [13]	Nigeria	High Risk Women
2004-2007	Carraguard Gel	Population Council Carraguard Trial [14]	South Africa	Sexually Active Women
2005-2007	Cellulose Sulphate Gel	CONRAD CS Trial [15]	Benin, India, South Africa, Uganda	High Risk Women
2005-2008	Buffer Gel and 0.5% PRO2000 Gel	HTPN 035 [16]	Malawi, South Africa, Zambia, Zimbabwe, USA	Sexually Active Women
2005-2009	0.5% PRO2000 Gel	MDP301 [17]	South Africa, Zambia, Uganda, Tanzania	Sexually Active Women
2007-2009	1% Tenofovir Gel	CAPRISA 004 [18]	South Africa (KwaZulu-Natal)	Sexually Active Women
2009-2012	1% Tenofovir Gel, Tenofovir Disoproxil Fumarate Tablet (Truvada) and Emtricitabine/ Truvada	MTN003 (VOICE) [19]	South Africa, Uganda, Zimbabwe	Sexually Active Women
2011-Current	1% Tenofovir Gel	FACTS001 [20]	South Africa	Sexually Active Women

N-9=Nonoxynol-9, MDP=Microbicides Development Programme, VOICE = Vaginal and Oral Interventions to Control the Epidemic, FACTS=Follow-on African Consortium for Tenofovir Studies, CAPRISA=Centre for the AIDS Programme of Research In South Africa, MTN=Microbicides Trials Network, CS=Cellulose Sulphate, HTPN=HIV Prevention Trials Network

Strategies to reduce the pregnancy rates require an understanding of the incidence and the associated risk factors of pregnancy in the microbicide trials. Although pregnancy rates have been reported in the literature, there is currently no published systematic review and meta-analysis of the incidence rates of pregnancy in the microbicide trials. This systematic review and meta-analysis aims to estimate an overall incidence rate of pregnancy and the associated risk factors of pregnancy in the microbicide trials. 

### Objectives

The objectives were to determine the overall incidence and risk factors of pregnancy in vaginal microbicide trials for the prevention of heterosexual acquisition of HIV infection in women.

## Methods

The Cochrane methodology was applied in this review, where applicable. The protocol for this systematic review is not published.

### Criteria for considering studies for this review

All studies reporting pregnancy, its incidence and risk factors among women participating in vaginal microbicide trials for prevention of heterosexual acquisition of HIV infection were included in this review. Randomized controlled trials (RCTs), secondary analyses, and review articles of microbicide trials were eligible for inclusion in the review. The participants were sexually active non-pregnant HIV-negative women of reproductive age. 

### Types of outcome measures

The primary outcome was pregnancy incidence. The secondary outcomes were risk factors of pregnancy.

### Search methods for identification of studies

A comprehensive literature search for quantitative published studies reporting on pregnancy, its incidence or risk factors in vaginal microbicide trials for HIV prevention was carried out, irrespective of language, using publication dates extending from 01 January 1990 to 16 June 2013 since the first microbicide trial was reported in 1992 [7]. The following electronic databases were searched: PubMed/ MEDLINE, EMBASE, Web of Science, and Cochrane databases. The keywords in the search included ‘pregnancy’, ‘contraceptive use’, ‘microbicide’ and ‘HIV prevention trial’. The actual PubMed search strategy used is given below. 

((pregnancy) OR pregnancy[MeSH]) AND ((contraception) OR (contraceptive use) OR contraception[MeSH] OR contraceptive[MeSH]) AND (((microbicide) OR microbicide[MeSH]

)OR((HIV prevention trial) OR HIV prevention trial [MeSH]))

Limits: Date of publication -1990/01/01 to 2013/06/16

This search strategy was modified to search other electronic databases. The related citations in PubMed were also searched for potential studies. In addition, reference lists of microbicide trials, relevant review articles and included studies of this review were searched for potential studies that would have been missed by the electronic search. Authors of microbicide trials were also searched in PubMed to identify any other potential studies.

### Selection of studies

Two review authors, Alfred Musekiwa (AM) and Evans Muchiri (EM), independently screened the title and abstract results from the search and applied pre-piloted eligibility criteria to identify eligible studies. Full-text articles of potential studies were retrieved where at least one review author selected it for inclusion. The articles were scrutinized for multiple publications. Study authors were contacted where there was either missing information or where clarity was required. Disagreements between review authors were resolved through discussion.

### Data extraction and quality of studies

Using a pre-designed data extraction form, two review authors (AM and EM) extracted data on pregnancy and its risk factors. Incidence rates of pregnancy (per 100 woman-years) were extracted, together with the number of total participants and number of first pregnancies during the trial. With regards to risk factors, only significant (p<0.05) factors of pregnancy in multivariate analyses were extracted with their corresponding Adjusted Hazard Ratios (AHR) or Adjusted Odds Ratios (AOR), 95% confidence intervals (CI) and p-values. Risk factors which were significant only in univariate analyses were not considered due to their potential bias. Disagreements between review authors were resolved through discussion.

No assessment of quality of studies could be done because different study designs (RCTs, secondary studies and reviews) were included.

### Data synthesis and management

The incidence rates of pregnancy (per 100 woman-years) from the different studies were pooled in a meta-analysis. Random effects meta-analysis was used because heterogeneity was expected since the studies were done in different populations. The generic inverse-variance method of meta-analysis was applied to the incidence rates using STATA 12.0. Heterogeneity between studies was assessed through the inspection of the forest plot to identify any lack of overlapping of confidence intervals which would indicate heterogeneity, the Chi-square test to determine whether the heterogeneity was due to chance alone, and also the I-square statistic which gives a percentage of variation that is due to heterogeneity rather than chance alone [24]. The investigation of sources of heterogeneity was carried out using both sensitivity and subgroup analyses. Sensitivity analysis was conducted with respect to one microbicide trial that had an unusually high pregnancy incidence compared to the other trials. This involved examining the effect of excluding the trial from meta-analysis on the magnitude of heterogeneity and also on the overall pregnancy incidence. Subgroup analysis with respect to different types of microbicides involved subgrouping trials of the same microbicide and performing meta-analyses within these subgroups. This was done so as to determine whether there was significant heterogeneity within the subgroups. Meta-analysis results for the subgroups were then reported separately. An attempt to perform subgroup analysis with respect to different countries in which the trials were done was unsuccessful because some of the trials were multi-country trials. 

The risk factors of pregnancy were reported narratively. 

## Results

### Results of the search and description of studies

**Figure 1:**
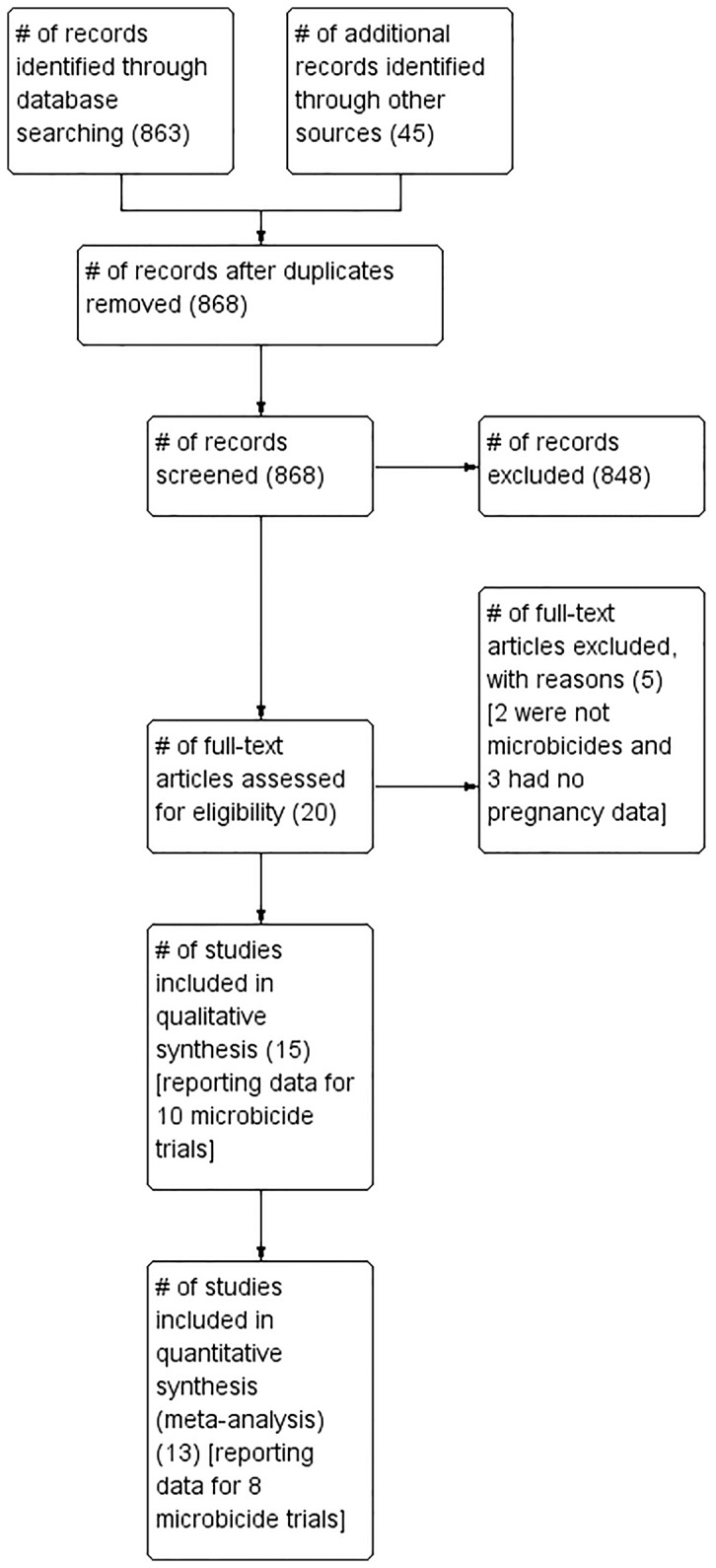
Study flow diagram.

The study flow diagram is shown in Figure 1. Electronic search of the available databases yielded 863 records. Checking the reference lists of review articles and selected studies identified 45 extra records. After excluding records that did not meet the inclusion criteria and duplicates, 20 records for which full-texts were retrieved remained. After reading the full-texts, a further 5 records were excluded because they either did not investigate a microbicide product [21,22] or did not report pregnancy data [6,7,9]. The remaining 15 studies reporting data from 10 microbicide trials were included in this systematic review [8,10-18,25-29]. Incidence rates for 8 microbicide trials were included in the meta-analysis because the incidence rates for 2 trials were not reported.

### Included studies

#### Participants, settings and interventions

The participant characteristics, settings and interventions for the ten microbicide trials included in this review are summarized in Table 2. The trials were conducted mainly in Sub-Saharan Africa (South Africa, Zimbabwe, Zambia, Malawi, Nigeria, Ghana, Uganda, Kenya, Cameroon, Tanzania, Benin, and Cote d’Ivoire). The studies also included trials conducted in USA, India, and Thailand. The participants were women aged above 18 years, except in some South African trials where women were enrolled from 16 years of age. Participants from two trials were female sex workers but the remaining trials described their participants as sexually active women recruited from reproductive health clinics, local market areas, bars, hostels, military barracks, colleges, hotels, shopping malls, churches, taxi ranks, truck stops, and other community venues. All the trials recruited from urban settings except the CAPRISA004 trial which had one of its two sites in a rural clinic in KwaZulu-Natal Province, South Africa. The type of microbicides in the included studies were nonoxynol-9 (N-9), cellulose sulphate (CS), carraguard, SAVVY (C31G), Buffer Gel, PRO 2000 and Tenofovir.

**Table 2:** A summary of characteristics of included studies.

**Microbicide Trial**	**Participants and settings**
N-9 Film/ Cameroon	1292 female sex workers aged 18-45 years
N-9 Gel/ South Africa, Thailand, Benin, Cotê d’Ivoire	892 female sex workers aged 16 years and above from clinic settings
FHI SAVVY/ Ghana	2142 women aged 18-35 years from high HIV transmission areas including markets, bars and hotels
FHI SAVVY/ Nigeria	2153 women aged 18-35 years from high HIV risk areas such as local market areas, bars, hostels, military barracks and colleges
FHI CS/ Nigeria	1644 women aged 18-35 years from high HIV risk areas such as bars, markets, and other common gatherings
Population Council Carraguard/ South Africa	6202 women aged 16 years and above from local health clinics, shopping malls, churches, taxi ranks, and other community venues
CONRAD CS/ Benin, India, South Africa, Uganda	1428 sexually active women aged 18 years and above attending community clinics, STI clinics and hospitals
HTPN035/ Malawi, South Africa, Zambia, Zimbabwe, USA	3101 sexually active women aged 18 years and above
MDP301/ South Africa, Tanzania, Uganda, Zambia	6,651 sexually active women aged 16 years and above enrolled from 13 clinic settings in South Africa, Tanzania, Uganda, and Zambia
CAPRISA004/ South Africa	889 sexually active women aged 18-40 years enrolled at an urban and rural area in KwaZulu-Natal, South Africa

N-9=Nonoxynol-9, MDP=Microbicides Development Programme, CAPRISA=Centre for the AIDS Programme of Research In South Africa, CS=Cellulose Sulphate, HTPN=HIV Prevention Trials Network, USA=United States of America.

#### Incidence rate of pregnancy

**Figure 2:**
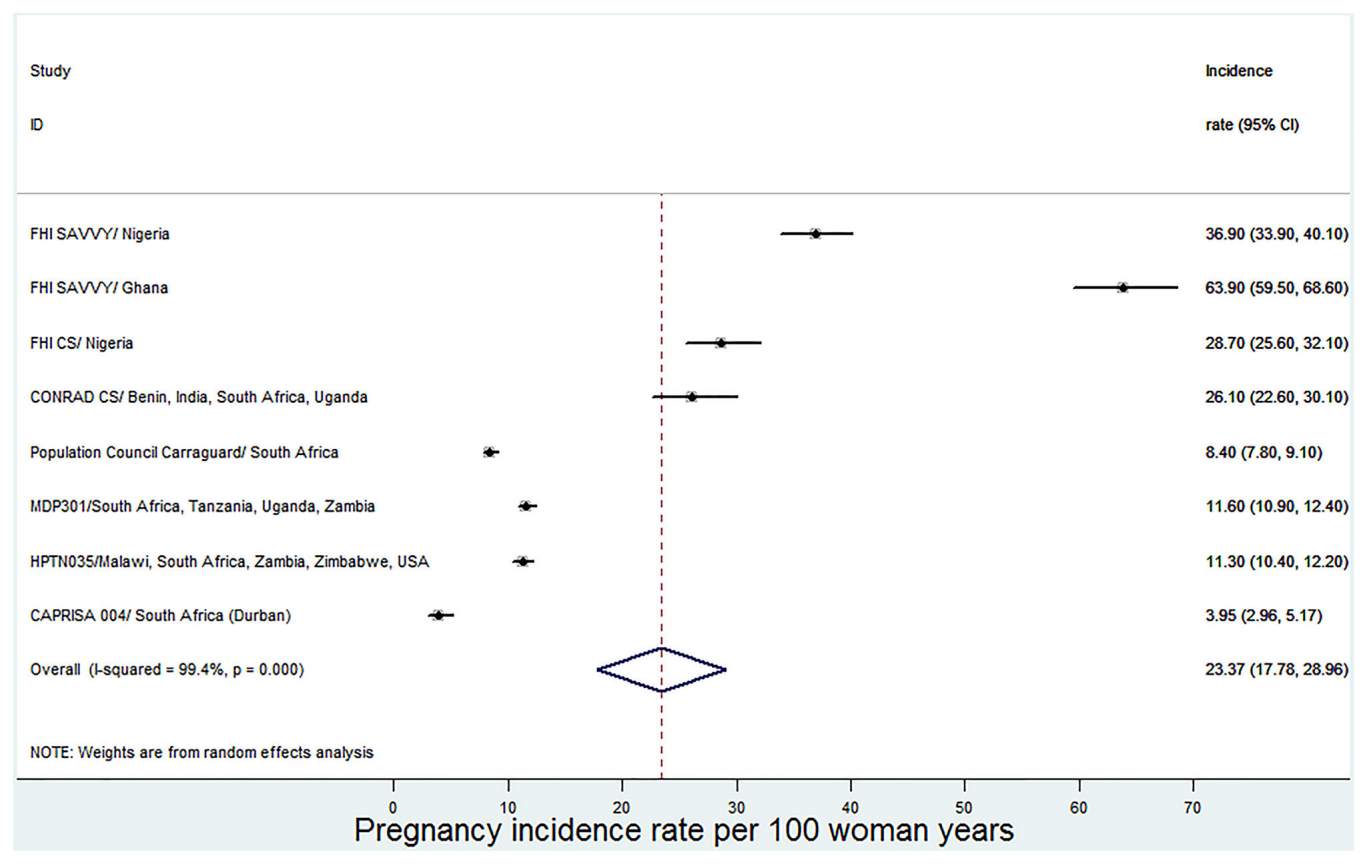
Forest plot for the meta-analysis of incidence rates of pregnancy (per 100 woman-years) in microbicide trials.

The incidence rate of pregnancy per 100 woman-years and the percentage of pregnant women from the included studies are given in Table 3. For some microbicide trials, there are two or more studies reporting the same pregnancy data. From a total of 27,384 participants included in this review, 4,107 participants (15.0%) fell pregnant during the trial periods (Table 3). The incidence rates of pregnancy (per 100 woman-years) from 8 microbicide trials were pooled in a random-effects meta-analysis. The rates ranged widely from 3.95 to 63.9 pregnancies per 100 woman-years. The meta-analysis yielded a high incidence rate of 23.37 (95%CI: 17.78 to 28.96) pregnancies per 100 woman-years (Figure 2). However, the Chi-square test (Chi^2^=1180.69, degrees of freedom (df) =7, p<0.0001) and the I-square statistic (I^2^=99.4%, p<0.0001) indicated significant heterogeneity between the trials. The forest plot (Figure 2) also showed lack of overlap of confidence intervals, which is an indication of high heterogeneity. 

**Table 3:** Pregnancy data for included studies.

**Microbicide Trial**	**Included study1**	**Incidence rate of pregnancy per 100 woman-years (95%CI)**	**No. pregnant/Total (%)**
N-9 Film/ Cameroon	Roddy 1998 [8]	NR	5/941 (0.5%)
N-9 Gel/ South Africa, Thailand, Benin, Cotê d’Ivoire	Van Damme 2002 [10]	NR	10/892 (1.1%)
FHI SAVVY/ Ghana	Peterson 2007 [11], Halpern 2011 [25], Sibeko 2012 [26]	63.9 (59.5, 68.6)	769/2038 (26.5%)
FHI SAVVY/ Nigeria	Feldblum 2008 [12], Halpern 2011 [25], Sibeko 2012 [26]	36.9 (33.9, 40.1)	552/2082 (26.5%)
FHI CS/ Nigeria	Halpern 2008 [13], Halpern 2011 [25], Sibeko 2012 [26]	28.7 (25.6, 32.1)	308/1526 (20.5%)
Population Council Carraguard/ South Africa	Skoler-Karpoff 2008 [14], Sibeko 2012 [26]	8.4 (7.8, 9.1)	682/6005 (11.4%)
CONRAD CS/ Benin, India, South Africa, Uganda	Van Damme 2008 [15], Halpern 2011 [25], Sibeko 2012 [26]	26.1 (22.6, 30.1)	197/1122 (27.0%)
HTPN035/ Malawi, South Africa, Zambia, Zimbabwe, USA	Abdool Karim SS 2011 [16], Sibeko 2012 [26]	11.3 (10.4, 12.2)	613/3050 (20.1%)
MDP301/ South Africa, Tanzania, Uganda, Zambia	McCommark 2010 [17], Sibeko 2012 [26], Schreiber 2011 [27]	11.6 (10.9, 12.4)	917/8859 (10.4%)
CAPRISA004/ South Africa	Abdool Karim Q 2010 [18], Sibeko 2012 [26], Ramjee 2012 [28] , Sibeko 2011 [29]	3.95 (2.96, 5.17)	54/889 (6.1%)

1=Some trials were reported by more than one study, NR=Not Reported, N-9=Nonoxynol-9, MDP=Microbicides Development Programme, CAPRISA=Centre for the AIDS Programme of Research In South Africa, CS=Cellulose Sulphate, HTPN=HIV Prevention Trials Network

#### Investigation of sources of heterogeneity

**Figure 3:**
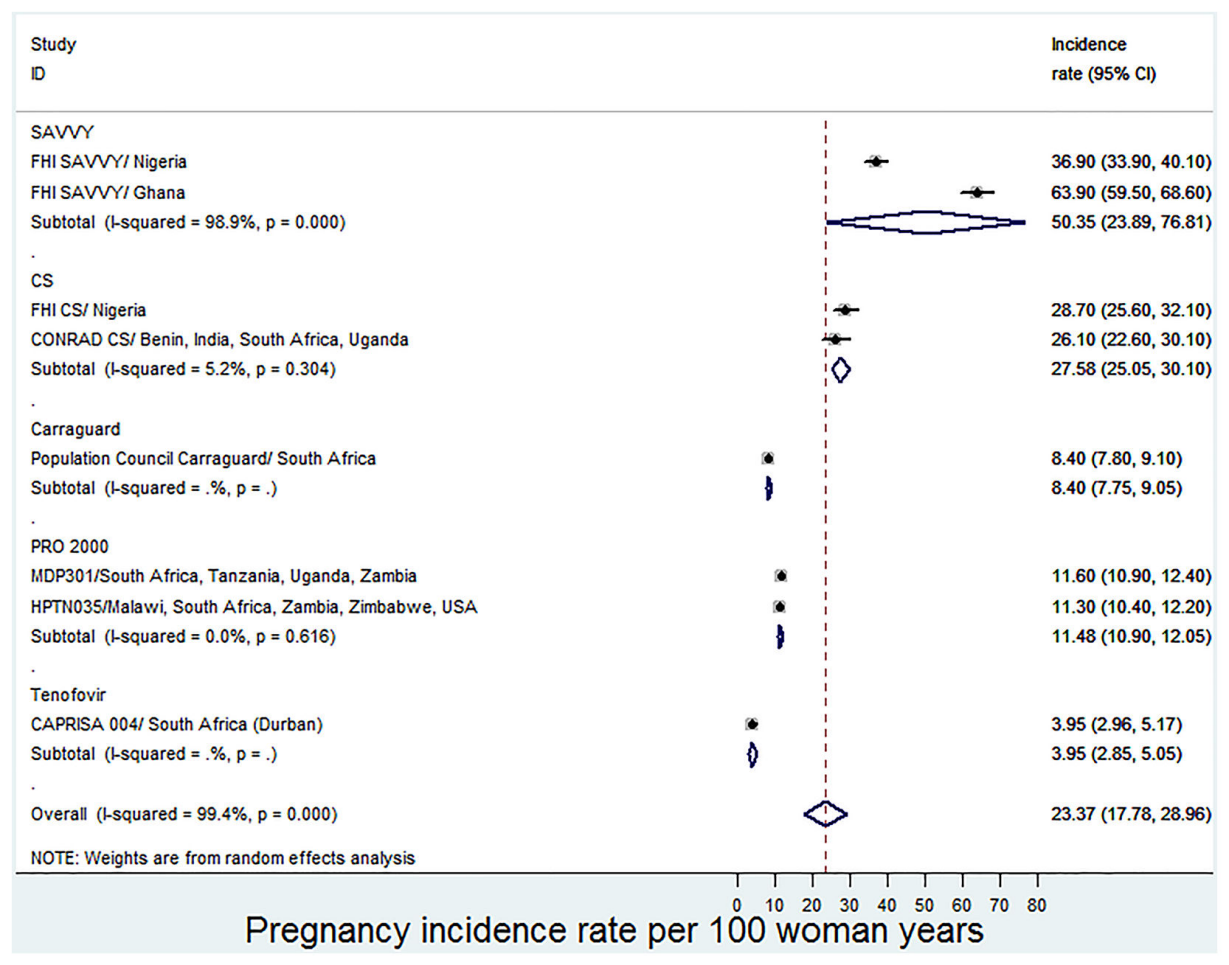
Forest plot for the meta-analysis of incidence rates of pregnancy (per 100 woman-years) in microbicide trials with subgroup analysis.

The forest plot shown in Figure 2 clearly indicate that the pregnancy incidence rate for the FHI SAVVY/ Ghana trial is extremely higher compared to others. A sensitivity analysis was carried out by excluding this trial from the meta-analysis and this resulted in a reduction in the overall pregnancy incidence rate to 17.76 (95%CI: 13.29 to 22.23) pregnancies per 100 woman-years but heterogeneity remained significant (I^2^=99.1%, p<0.0001). Subgroup analyses with respect to the type of microbicide yielded significant heterogeneity only for the SAVVY Group (I^2^=98.9%, p<0.0001, Figure 3) but no significant heterogeneity with respect to all the other subgroups (Figure 3). Since there was no significant heterogeneity within all the remaining subgroups, the type of microbicide may probably be a source of heterogeneity.

The pooled pregnancy incidence rates (per 100 woman-years) for the different types of microbicides (subgroups) ranged from 3.95 for Tenofovir (95%CI: 2.85 to 5.05), then 8.40 for Carraguard (95%CI: 7.75 to 9.05), then 11.48 for PRO 2000 (95%CI: 10.90 to 12.05), then 27.58 for CS (95%CI: 25.05 to 30.10), up to 50.35 for SAVVY (95%CI: 23.89 to 76.81) (Figure 3).

An attempt to compare trials with pregnancy rate higher than 20 pregnacies per 100 woman-years (SAVVY, CS) and those with less than 20 pregnancies per 100 woman-years (Carraguard, HPTN035, MDP301, CAPRISA004) with respect to demographic factors (age and marital status) and sexual behavioural characteristics (sexual acts per week and contraception) resulted in no apparent differences, although some of the data were not reported (Table 4). 

**Table 4:** Baseline demographic characteristics of women participants as reported in included trials.

**Microbicide Trial**	**Pregnancy incidence rate per 100 woman-years**	**Average age (years)**	**% Married**	**Average number of sex acts per week**	**% on contraception**
FHI SAVVY/ Ghana	>20	22.7	11.7	9.2	61.8
FHI SAVVY/ Nigeria	>20	23.6	14.0	10.4	93.9
FHI CS/ Nigeria	>20	23.4	5.0	6.0	79.9
CONRAD CS/ Benin, India, South Africa, Uganda	>20	29	22.9	4.0	44.4
Population Council Carraguard/ South Africa	<20	30.7	31.0	2.0	77.0
HTPN035/ Malawi, South Africa, Zambia, Zimbabwe, USA	<20	26.3	63	2.9	68.0
MDP301/ South Africa, Tanzania, Uganda, Zambia	<20	NR	NR	NR	56.0
CAPRISA004/ South Africa	<20	23.9	5.6	NR	100

NR=Not Reported, MDP=Microbicides Development Programme, CAPRISA=Centre for the AIDS Programme of Research In South Africa, CS=Cellulose Sulphate, HTPN=HIV Prevention Trials Network, USA=United States of America

#### Risk factors of pregnancy

Only 4 out of the 15 included studies reported on the risk factors associated with pregnancy. Multivariate Cox regression results from one study using data from 4 microbicide trials showed that hormonal injectable (AHR 0.45, 95%CI: 0.33 to 0.61) and IUD or implants or sterilization (AHR 0.21, 95%CI: 0.09 to 0.48), compared with none or other or emergency contraceptive methods, at baseline, were associated with reduced rates of pregnancy incidence. Older age (AHR 0.90, 95%CI: 0.89 to 0.92), more years of education (AHR 0.97, 95%CI: 0.96 to 0.99), and condom use (AHR 0.88, 95%CI: 0.79 to 0.99) were also associated with lower pregnancy [25]. Multivariate logistic regression from another study also showed that older age (AOR 0.88, 95%CI: 0.79 to 0.98, p=0.02) was associated with a reduction in pregnancy incidence [27]. Multivariate Cox regression from another study found that hormonal contraceptive use both at baseline (AHR 0.34, 95%CI: 0.24 to 0.48) and during study (AHR 0.36, 95%CI: 0.27 to 0.48) was associated with reduced pregnancy rate [28].

On the other hand, living with a man (AHR 1.20, 95%CI: 1.03 to 1.40), history of pregnancy (AHR 1.32, 95%CI: 1.15 to 1.51), higher number of pregnancies (AHR 1.05, 95%CI: 1.01 to 1.10), higher number of vaginal deliveries (AHR 1.19, 95%CI: 1.12 to 1.27), and higher number of unprotected sex acts (AHR 1.03, 95%CI: 1.01 to 1.05) were all associated with higher pregnancy [25]. Self and partner desire to have a baby in future (AOR 4.95, 95%CI: 1.35 to 18.09, p=0.02) was also associated with higher pregnancy [27]. Oral contraceptive use at baseline (AHR 15.91, 95%CI: 8.03 to 31.52, p<0.0001) and inconsistent condom use in the past 30 days (AHR 2.05, 95%CI: 1.04 to 4.04, p=0.04) were associated with higher rates of pregnancy incidence [29].

## Discussion

The objectives of this systematic review were to determine the overall incidence rate of pregnancy and the associated risk factors in vaginal microbicide trials for the prevention of sexual acquisition of HIV. A comprehensive and detailed search for studies through electronic databases and checking of references of both included studies and relevant articles yielded a total of 15 included studies reporting data from 10 vaginal microbicide trials. 

The incidence rate of pregnancy for 8 microbicide trials included in the meta-analysis ranged widely from 3.95 to 63.9 pregnancies per 100 woman-years. The lowest rate of pregnancy was observed in the CAPRISA 004 trial in South Africa (Durban) and the highest rate was observed in the FHI SAVVY/ Ghana trial. The lowest value for the CAPRISA 004 trial can be attributed to the trial’s comprehensive contraceptive curriculum which was aimed at reducing pregnancy rate [29]. The highest rate of pregnancy from the FHI SAVVY/ Ghana trial may be attributed to the fact that the participants were the youngest in terms of their average age. A meta-analysis of the pregnancy rates yielded a high overall rate of 23.37 (95%CI: 17.78 to 28.96) pregnancies per 100 woman-years, although significant heterogeneity was detected between the studies. The overall pregnancy rate translates to 23 pregnancies per 100 women followed up over a period of one year. 

The factors that were found to reduce pregnancy included effective contraceptive methods such as hormonal injectable and IUD or implants or sterilization, older age, more years of education and condom use. On the other hand, the factors that were associated with higher pregnancy were living with a man, history of pregnancy, self and partner desire to have a baby in future, oral contraceptive use, increased number of unprotected sexual acts and inconsistent use of condoms.

This systematic review and meta-analysis has a number of strengths. Firstly, this is the first systematic review to address the meta-analysis of incidence rates of pregnancy in vaginal microbicide trials for the prevention of sexual acquisition of HIV. There is currently a published review with no quantitative synthesis [26]. The search for studies was comprehensive using a detailed search strategy with Medical Search Headings (MeSH) terms. Reference lists of relevant articles were also scanned to identify any eligible studies that could have been missed by the electronic searches. It is unlikely that any studies meeting the inclusion criteria could have been missed. The process of selecting studies and data extraction were done independently by two review authors and all authors checked the accuracy of the data extractions and analyses. The very high sample size of 27,384 participants gives this review more strength and statistical power. This review also extracted information on risk factors for pregnancy in these microbicide trials. Although the included studies could not be appraised for quality because of their different study designs (secondary analyses, randomized controlled trials, and reviews of microbicide trials), it was judged that the statistical methods applied in the included studies, mainly multivariate Cox regression, was the most appropriate method for determining risk factors associated with pregnancy incidence. 

With regards to limitations, heterogeneity in the meta-analysis limits the strength of the evidence of high pregnancy rate in microbicide trials. Further analysis through sensitivity and subgroup analyses showed that the type of microbicide may probably be a source of heterogeneity. The type of microbicide can be taken as representing the different study protocols with more strict requirements for effective contraception in later trials. The use of the random-effects model of meta-analysis, instead of the fixed effects model, was an attempt to attenuate the effect of heterogeneity on the meta-analysis results. Heterogeneity between results is, however, common in meta-analyses of incidence rates [30]. And although we detected heterogeneity, it was clear that the majority of the reported incidence rates were high. The second limitation is that this systematic review does not quantify the effect that the pregnancy rates had on the total person-years for the trials or the attrition rates of the trials. The other limitation was that not all included studies reported the risk factors associated with pregnancy incidence and therefore, no meta-analysis of results for these risk factors could be conducted. Another limitation was that the pregnancy results for six microbicide trials were not reported either because they were never published [7-10] or are still yet to be published [19,20]. 

The results of this systematic review and meta-analysis showing high rates of pregnancy in vaginal microbicide trials for the prevention of sexual acquisition of HIV agree with other studies [21,22]. 

### Author’s conclusions

#### Implications for practice

The incidence rate of pregnancy in vaginal microbicide trials for the prevention of sexual acquisition of HIV infection is very high and this impacts heavily on the sample size and power of these trials to detect an intervention effect at the analysis stage. Provision for dealing with potential attrition at the analysis stage should be put in place as part of the protocol. Strategies to reduce these pregnancy rates are urgently required. Counseling and provision of effective contraceptive methods should continue to be recommended. Selection criteria of participants who are less likely to fall pregnant can reduce pregnancy rates. 

#### Implications for research

Qualitative studies aimed at understanding the factors underlying the reasons why women participants fall pregnant during microbicide trials, despite the provision of counseling and effective contraception, are required.

## Supporting Information

Checklist S1 **PRISMA Checklist.**
(DOC)
